# Impact of mode of offer of self-sampling to people overdue cervical screening on screening participation: a randomised controlled trial

**DOI:** 10.1016/j.eclinm.2025.103357

**Published:** 2025-07-28

**Authors:** Anita W.W. Lim, Rebecca Landy, Jane Rigney, Bernard North, Peter D. Sasieni

**Affiliations:** a Wolfson Institute of Population Health, Queen Mary University of London, London, UK; b School of Cancer and Pharmaceutical Sciences, Faculty of Life Sciences and Medicine, King's College London, London, SE1 9RT, UK; c Division of Cancer Epidemiology and Genetics, National Cancer Institute, National Institutes of Health, Department of Health and Human Services, Bethesda, MD, USA

**Keywords:** Self-sampling, Cervical cancer screening, Human papillomavirus

## Abstract

**Background:**

Offering self-sampling to non-attenders increases cervical screening uptake, but the optimal approach for offering kits remains unclear.

**Methods:**

Randomised controlled trial offering self-sampling. 13 GP (general practitioner) practices were randomised (1:1) to flagging women ≥6-months overdue cervical screening so that they could be offered a self-sampling kit if they attended their GP for any reason (N = 6080 women), or no opportunistic offer (N = 6577 women). Additionally, never screened women and those overdue screening by 15- or 27-months were individually randomised (2:1:1) to usual care (no systematic offer), a letter inviting them to order a kit (letter), or being sent a self-sampling kit (kit). The study ran from April 2019 to March 2020. The primary outcome was returning a self-sampling kit, and the secondary outcome was any cervical screening. The International Standard Randomised Controlled Trial Number (ISRCTN) is 23940319.

**Findings:**

In opportunistic offer practices, 342 (5.6%) returned a self-sample compared with 1.9% (123/6577) in practices not randomised to opportunistic offering (adjusted risk difference 4.4% (95% CI: 2.8%–6.0%)). Half (234/449) of women offered self-sampling opportunistically returned a sample. Among 6400 women individually randomised to no systematic offer vs letter vs kit, 1.7% (54/3197), 4.8% (76/1587; difference relative to no systematic offer 3.1% 95% CI: 2.0–4.2%) and 12.3% (198/1616; difference relative to no systematic offer 10.5%, 95% CI: 8.9–12.2%), returned a self-sample (the primary outcome), respectively. These observed differences were maintained in the secondary outcome, any cervical screening. No adverse effects were reported.

**Interpretation:**

In-person offers were most effective, but only a small proportion of non-attenders received such an offer. Postal invitations without a kit were less effective. The secondary outcome suggests those screened by self-sampling would not have been screened otherwise and contribute to increased screening coverage.

**Funding:**

10.13039/501100000289Cancer Research UKC8162/A16892 to PS (consumables), C8162/A29083 to PS (JR, AL), C8162/A25356 to PS (BN); 10.13039/501100021701National Institute for Health Research Clinical Research Network (NIHR CRN) Central Portfolio Management System (CPMS) ID: 36156 (AL); Intramural Research Program, 10.13039/100000054Division of Cancer Epidemiology and Genetics, National Cancer Institute (RL).

**Research in context**

**Evidence before this study**

Before the trial began, we searched PubMed for articles published between 1 January 2000 and 1 January 2019 using the terms (“self-sampling” OR “self-sample” OR “self-collect”) AND (“HPV” OR “human papillomavirus” OR “cervical cancer, screening for cancer, HPV”). Studies have shown that sending self-sampling kits directly to underscreened women is more effective than sending a letter inviting women to order a kit. Results are variable but, in several countries, including the UK, response rates to sending out kits have been disappointing. Some studies suggest that in-person offers of kits to underscreened women attending primary care for any reason can be more effective, but this has not been established rigorously. A recent study showed that in-person opportunistic offering of self-samples is feasible at scale. One study suggested that the take-up of self-sampling is at the expense of regular screening and that sending kits in the post did not result in an increase in screening coverage.

**Added value of this study**

This pragmatic randomised controlled trial demonstrates the impact of three different approaches to offering self-sampling to women at least six months overdue cervical screening. It demonstrates that the impact of a letter without a kit is limited in the UK, and that about one in ten of those sent a kit returned an adequate sample; by contrast, approximately half of those offered a kit in primary care returned a self-sample. Secondary analysis of the proportions screened by any route showed that the offer of self-sampling resulted in more people overdue screening being screened, not just providing a self-sample.

**Implications of all the available evidence**

Opportunistic in-person offering of self-sampling in primary care to individuals at least 6 months overdue screening elicits the highest uptake of the kit distribution approaches with about half of those who are offered self-sampling participating in cervical screening and is effective in increasing coverage. However, this intervention only resulted in a minority of those overdue screening receiving such an offer over the course of a year. Supplementation with sending kits to those at least 15 months overdue screening will result in a large increase in screening coverage. Where poor coverage is an issue, in-person offers of self-sampling to those consulting in primary care and the systematic sending-out of kits should be considered as an effective way to boost participation in those overdue screening. Any such implementation should be monitored to ensure it is effective.

## Introduction

Organised cervical screening can prevent over 75% of cervical cancers cases (and an even higher proportion of deaths) and is estimated to have prevented 60% of cancers in England.^1^ Despite (or possibly because of) this success, attendance rates have been falling for years, which limits its population impact.^2^ Speculum examination is a major deterrent due to the associated physical and emotional discomfort.^3^^,^^4^ Human papillomavirus (HPV) testing has enabled a step-change for cervical screening in the form of self-sampling. Self-sampling provides a convenient and less invasive option with comparable sensitivity to clinician-taken samples for the detection of high-grade cervical disease.^5^ Several countries have already made self-sampling available for screening non-attenders.^6^ However, evidence from a meta-analysis and early adopter countries show heterogeneity in uptake between countries and method of kit offer.^5^ Therefore, although self-sampling has powerful disruptive potential, identifying the locally-appropriate strategy for kit distribution is essential. The most widely studied strategies are direct mailing of kits to women's homes and sending letters inviting women to order kits. In-person offers in GP primary care have been less studied but elicits the highest uptake.^7^^,^^8^ Despite heterogeneity, a consistent finding is that letters including a kit obtain a greater response than letters inviting women to order a kit.^7^

Self-sampling has recently been recommended for underscreened women in the UK (https://www.gov.uk/government/news/home-testing-kits-for-lifesaving-checks-against-cervical-cancer); plans are underway in England for a rollout but details are as yet unknown. UK studies^9^^,^^10^ of direct mailing of kits have reported disappointing uptake (6%–8%), particularly since similar studies in other European countries achieved 30–40% uptake.^11^, ^12^, ^13^, ^14^ Moreover, the associated kit attrition rates of over 90% is untenable for most screening programmes and the environment. The UK's National Health Service (NHS) provides a conduit for reaching non-attendees via primary care. An audit of electronic records in England found that 30% of cervical screening non-attenders consulted their GP at least once over three months, and 60% over one year.^15^ We tested this in a pilot study in East London^16^ in which non-attenders were offered self-sampling opportunistically when they consulted GP primary care for any reason. This resulted in 68% accepting a kit, and 45% returning a self-sample.

The aims of the present study were to explore different approaches for offering self-sampling to non-attenders, and to understand their comparative efficacy. We conducted a pragmatic randomised controlled trial evaluating: i) opportunistically personally offering self-sampling kits to non-attenders consulting primary care for any reason; ii) direct mailing of kits to non-attenders; and iii) sending a letter inviting non-attenders to order a kit.

## Methods

### Ethics

Ethical approval for the study was granted by the London–Brighton & Sussex Research Ethics Committee (REC) (ref 17/LO/1655). Everyone who returned a self-sample had to include a lab request form which informed them that by returning the self-sample they were consenting to take part in the study. This study was performed in accordance with the Declaration of Helsinki.

### Cervical screening in England

In England, the NHS provides free-of-charge cervical screening. People eligible for screening (i.e., women with no record of a hysterectomy and others with a cervix) are identified in a national database and are sent invitation and reminder letters via a centralised system. Invitations are sent three-yearly to individuals aged 25–49 years and five-yearly to individuals aged 50–64 years. A single reminder is sent three months after invitation to those who have not attended.

This pragmatic randomised controlled trial was conducted in the London Borough of Hounslow, an ethnically diverse area (50% born outside the UK, 37% Asian, 7% Black^17^) with low cervical screening coverage (61.6% in 2022–2023). ^18^ Non-attenders were by definition at least 6 months overdue screening according to their GP records. The trial aimed to recruit 12–13 GP practices. All practices within the Hounslow Clinical Commissioning Group (CCG, an organisation responsible for planning and commissioning healthcare services locally) were eligible. The trial was presented at CCG-wide meetings and email distribution lists, and several practices were approached by the local clinical research network (CRN). Interested practices contacted the study team or CRN.

### Randomisation and masking

Figure S1 provides an overview of the timing of the trial interventions relative to the call/recall invitations in the English screening programme. The trial used both cluster randomisation and individual-level randomisation to assess the self-sampling interventions.

#### Cluster randomisation

The first aim was to evaluate the impact of opportunistically offering women overdue screening a self-sampling kit if they attended their GP practice for any reason. GP practices were cluster randomised 1:1 to “opportunistic offer” (the intervention) or “no opportunistic offer” (i.e., usual care). GP practices were randomised in blocks of two using computer-generated random numbers. Study posters outlining the study were provided to GP practices in the opportunistic offer arm to display.

The cluster randomisation comprised an open cohort. Eligible women could become ineligible (if they were screened or moved practice), and vice-versa (by becoming 6 months overdue or joining the practice). Electronic GP records were used (i) before and during the study to assess eligibility, and (ii) after the study to collect screening history, opportunistic kit offers (which were entered into electronic GP records when they occurred) and follow up of those testing HPV positive. Self-sample test dates and results were provided by the laboratory.

#### Individual randomisation

We additionally wanted to know what proportion of women overdue screening would return a self-sample kit if they were mailed a kit or a letter inviting them to order a kit. Therefore women aged 25–64 years from both the opportunistic offer and no opportunistic offer GP practices who (i) had never been screened, (ii) became 15-months overdue cervical screening during the study or (iii) were >15 months overdue at the start of the study and became 27-months overdue during the study, were individually randomised 2:1:1 to (a) usual care (no systematic offer), (b) mailing a self-sampling kit (sent kit), or (c) mailing a letter inviting them to order a kit (sent letter). Women randomised to no systematic offer for the individual-level randomisation did not receive any kit offer sent to their home; instead they would just receive the usual invitation letters for clinician screening after the usual interval, or anything else the practice would normally do to follow up with lapsed women. For example, some practices in England call women who are overdue screening to ask them to book an appointment, but this is not mandatory and is very variable between practices.

Individual-level randomisation was performed for women in both the opportunistic offer and no opportunistic offer arms so that we could determine whether there was an interaction between the interventions. Individual-level randomisation was stratified by GP practice, with randomisation performed among individuals who were newly eligible for individual randomisation once a month. The 15-month timepoint was selected as it is one year after the reminder letter. The 27-month timepoint acted as a “catch-up” for non-attenders already >15 months overdue at the study start. GP practices randomised women at their practice using a randomisation programme created in Microsoft Excel. Unless a woman received a kit or letter in the mail, they would not know they had been individually randomised.

GPs in both arms were blinded to the woman's individual-level randomisation category, therefore randomisation should not have impacted who was offered a kit opportunistically.

### Study interventions

At opportunistic offer practices, the primary care record software systems (EMIS) were programmed to “flag” eligible women by showing an on-screen message when the record of an eligible woman was opened. For eligible women who attended a consultation during the study period for any reason, GPs, practice nurses, and healthcare practitioners were encouraged to offer self-sampling kits during the consultation. However, the offer of a self-sampling kit was made on a case-by-case basis at the discretion of the healthcare professional consulted (e.g., self-sampling kits would not be offered to women who are terminally ill). At the end of their consultation, women were given a brief explanation about the study and self-sampling, using a template in EMIS with suggested text. Once eligibility was confirmed, women who were interested were given a self-sampling kit and had the option of collecting their sample in the clinic bathroom or at home. Samples collected at the GP practice were posted to the laboratory by the GP practice staff. At practices not randomised to opportunistic offering, there were no “flags” and no opportunistic offering of kits.

Mailed kits (sent to those randomised to the ‘sent kit’ arm, and to those ‘sent letter’ participants who ordered a kit) comprised a FLOQSwab 552C.80 (Copan Italia), an invitation letter, a patient information leaflet, a self-sample instruction sheet, an HPV information leaflet, a laboratory consent form and a freepost box pre-addressed to the laboratory. Letters invited women to order a kit online or by returning a mail order form in a freepost envelope. Opportunistic kits comprised the same components as mailed kits, excluding the invitation letter.

### Clinical management

Test results were mailed to participants and copied to their GP. HPV-negative women were advised in their letter that no further action was required and to attend screening when next invited. However, this result did not affect when the screening programme next invited them. Women with an inadequate sample (usually due to insufficient DNA) were sent another self-sampling kit with an accompanying letter explaining that their sample was inadequate and asking them to collect another sample. Women who tested HPV positive were advised in their results letter to have a clinician-taken follow-up test in the form of routine cervical screening. When the study started, routine screening used liquid-based cytology with HPV testing of the residual sample in those with low-grade abnormalities. In December 2019, screening switched to primary HPV testing.

### Study period & data collection

The trial initially opened on 24th April 2018 with staggered site start dates, however on 25th May 2018 several issues were discovered with the self-sampling kits that had been provided to the sites leading to a study halt on 29th May 2018. Specifically, the self-sampling kits supplied by the laboratory were missing the instruction sheet and HPV information leaflet. The four sites which had been initiated were closed while the issue was investigated. Given the quality control issues, after a full investigation, the decision was made to re-contract with a different testing laboratory who were more experienced with producing self-sampling kits and had robust QC procedures. In January 2019, the research group moved universities which led to further delays in re-starting the trial with the changes in sponsorship, contract negotiations and other administrative changes.

The trial re-started in March 2019 following delays due to contracting with a new laboratory and re-location of the research group. GP practices entered the study between 09th April and 22nd May 2019. Due to COVID-19 restrictions, sites were informed on 31st March 2020 that the study was closed to recruitment. Self-sample kits were analysed until 30th June 2020. GP records were extracted for eligible women up until 24th November 2021. Only anonymous data were collected for women who were randomised but did not return a self-sample, since they had not given consent.

### Sample transport & HPV testing

Samples were transported dry at ambient temperature. HPV testing was performed by Preventx Ltd (Sheffield, UK) using Roche cobas® 6800. Self-samples were resuspended in 4.3 ml Roche cobas PCR media upon arrival at the lab. All samples were vortexed for 30 seconds using a multi-sample vortexer. Samples were stored at ambient temperature until the next vortex run.

The cobas® 6800 Human Papillomavirus (HPV) test is a qualitative in vitro test for the detection of HPV in patient specimens. The test utilises amplification of target DNA by the Polymerase Chain Reaction (PCR) and nucleic acid hybridisation for the detection of 14 high-risk (HR) HPV types in a single analysis. The test specifically identifies (types) HPV16 and HPV18 while concurrently detecting the rest of the high-risk types (31, 33, 35, 39, 45, 51, 52, 56, 58, 59, 66, and 68).

### Sample size

Our goal was to estimate the magnitude of the effects with reasonable accuracy rather than on hypothesis testing.

With 12 GP practices randomised, there would be ∼6000 eligible women in each cluster-arm. Based on a previous 12-month study, in which 9% of the 60% of women in the opportunistic offer arm who had a primary care appointment returned a self-sampling kit,^16^ we expect 324 (5.4%) women to return a self-sample in response to an opportunistic offer. Ignoring intraclass correlation, this proportion could be estimated with a 95% confidence interval of approximately ±0.6%.

With 12,000 women overdue screening, we expected to individually randomise 6000–8000: 1500–2000 to each of the two individual-level intervention arms. Assuming 12% of those sent a kit directly return a sample, the expected 95% confidence interval would be ±1.5%. If 7% of those offered a kit by post return a sample, this could be estimated with ±1.3%.

### Statistics

The primary endpoint was return of a self-sampling kit within 6 months of offer. The proportion of eligible women (at least 6 months overdue screening at some point in the trial) meeting this endpoint was estimated separately by cluster randomisation arm, by individual randomisation arm and for the subgroup of women known to have been offered a kit opportunistically. We also estimated the contrasts (absolute differences in risk) associated with the interventions: opportunistic offer versus no opportunistic offer; letter versus no systematic offer; and kit vs letter (See below for model fitting).

Results are reported overall, and by age, ethnicity, and screening history. Screening history was defined as late (6–24 months overdue), very late (>24 months overdue, but screened previously) or never screened. Women aged <28 years who had never been screened were classified as ‘late’.

Since with only 13 clusters, randomisation might not result in equal distributions of age, ethnicity and screening history in the two arms, we used mixed-effects logistic regression with fixed effects for cluster randomisation, age, ethnicity and screening history, and random intercepts for GP practice. The marginal difference in uptake between arms was estimated using the margins command in Stata (which may not be the same as the crude difference in proportions). Eleven women with unknown screening history were dropped from these analyses. We additionally modelled interactions between each of age, ethnicity and screening history with cluster randomisation, to evaluate whether opportunistic offer performed equally among all subgroups (defined by age, ethnicity and screening history). For comparisons based on the individual-level randomisation, we used the difference in proportions. No adjustment was made for GP practice, since each GP practice randomised women on an individual-level.

We conducted several sensitivity analyses. During the study, a small proportion of women were randomised twice on an individual-level, due to a software programming error which allowed non-responders to be re-randomised the following month. The majority of women were either randomised to usual care at least once, or to the same intervention twice, though 41 women were randomised to be sent both a letter and a kit. In the main analyses, these women were analysed per initial randomisation. In the sensitivity analyses, we (i) used the most intensive intervention (usual care < sent letter < sent kit), and (ii) excluded these women. Additionally, we considered self-sample kits returned within 4 months, within 8 months and until 31 January 2021 (the latter to allow for the impact of COVID restrictions).

Secondary analyses included (i) assessing via logistic regression whether GP practice, age, ethnicity or screening history impacted the probability of being opportunistically offered a self-sampling kit; (ii) the proportion of women offered a kit opportunistically who accepted a kit; (iii) compliance to follow up–the proportion of self-sample positive participants who either had a clinician-taken sample or attended colposcopy within 6 months (from the date the self-sample result was sent); (iv) the proportion of women who tested HPV-positive on a self-sampling kit who have CIN2+ recorded in their GP records; and (v) the mode of kit order for women sent a letter inviting them to order a kit.

Although not pre-specified, we also considered the secondary endpoint “screened by any method (self-sample or routine screening) during the study” to explore the increase in screening uptake overall since some women who returned a self-sample might have attended routine screening had they not been offered self-sampling.

Women who changed GP practice during the trial were analysed based on their practice at randomisation. Thus, some women randomised to no opportunistic offer at the GP-level may have received a self-sampling offer by moving to a practice randomised to opportunistic offering.

We followed the CONSORT reporting guidelines, with the extension for pragmatic trials.^19^

### Role of the funding source

The funders had no role in the design and conduct of the study; in the collection, analysis, and interpretation of the data; preparation, review, or approval of the manuscript; and decision to submit the manuscript for publication. The authors alone are responsible for the views expressed in this article and they do not necessarily represent the views, decisions, or policies of the institutions with which they are affiliated.

## Results

### Study population

**Fig. 1:**
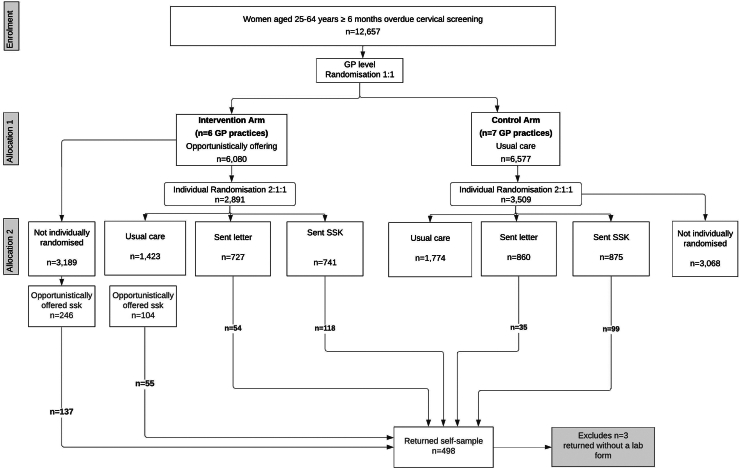
CONSORT figure.

Thirteen GP practices participated. Six were randomised to the opportunistic offer arm. Ineligible women (n = 142) who were individually randomised (88 had recently left the GP practice, 32 were not 6 months overdue screening, 4 were outside the age range, and 18 attended routine cervical screening after being randomised but before returning a sample) were excluded. After these exclusions, the opportunistic offer arm contained 6080 women eligible for an offer of self-sampling if they consulted their GP. The median number of eligible women in the 6 GP practices in the opportunistic offer arm was 943 (range: 658–1689). Of these, 2891 (47.5%) were individually randomised, 1423 to no systematic offer, 741 to being sent a kit, and 727 to being sent a letter (Fig. 1). Among GP practices assigned to no opportunistic offer, 6577 women aged 25–64 years were at least 6 months overdue screening. The median number of eligible women in the 7 GP practices in the no opportunistic offer arm was 807 (range: 297–2124). 3509 (53.3%) were individually randomised, 1774 to no systematic offer, 875 to be sent a self-sampling kit, and 860 to be sent a letter.

The characteristics of the study population are shown in Table 1. Almost three-quarters were aged <45 years, with 43% under 35. Of the 91% of women with a known ethnic background, 47% were White, 37% Asian, and 8% Black. Nearly half (42%) had never attended cervical screening, an additional 27% were over 2-years overdue screening. The number eligible per GP practice ranged from 297 to 2124. Due to differences in the populations that attend each GP practice, there were differences in the ages, ethnic backgrounds and time since last screen for participants by GP-level randomisation (Table 1, all p < 0.001).

**Table 1:** Demographic characteristics and screening history of eligible women, by cluster and individual-level randomisation.

Cluster randomisation	Opportunistic offer of self-sampling kit					No opportunistic offer of self-sampling kit					Total				
Individual-level randomisation	Not randomised	No systematic offer	Sent letter	Sent kit	Total	Not randomised	No systematic offer	Sent letter	Sent kit	Total	Not randomised	Usual care	Sent letter	Sent kit	Total
**Total**	3189	1423	727	741	6080	3068	1774	860	875	6577	6257	3197	1587	1616	12,657
**Age (years)**															
25–34	967 (30.3%)	743 (52.2%)	369 (50.8%)	385 (52.0%)	2464 (40.5%)	1079 (35.2%)	950 (53.6%)	458 (53.3%)	466 (53.3%)	2953 (44.9%)	2046 (32.7%)	1693 (53.0%)	827 (52.1%)	851 (52.7%)	5417 (42.8%)
35–44	1173 (36.8%)	388 (27.3%)	203 (27.9%)	202 (27.3%)	1966 (32.3%)	1045 (34.1%)	490 (27.6%)	232 (27.0%)	235 (26.9%)	2002 (30.4%)	2218 (35.4%)	878 (27.5%)	435 (27.4%)	437 (27.0%)	3968 (31.4%)
45–54	636 (19.9%)	184 (12.9%)	92 (12.7%)	98 (13.2%)	1010 (16.6%)	581 (18.9%)	188 (10.6%)	106 (12.3%)	108 (12.3%)	983 (14.9%)	1217 (19.5%)	372 (11.6%)	198 (12.5%)	206 (12.7%)	1993 (15.7%)
55–64	413 (13.0%)	108 (7.6%)	63 (8.7%)	56 (7.6%)	640 (10.5%)	363 (11.8%)	146 (8.2%)	64 (7.4%)	66 (7.5%)	639 (9.7%)	776 (12.4%)	254 (7.9%)	127 (8.0%)	122 (7.5%)	1279 (10.1%)
**Ethnic background**															
White	1706 (53.5%)	702 (49.3%)	364 (50.1%)	368 (49.7%)	3140 (51.6%)	1197 (39.0%)	581 (32.8%)	283 (32.9%)	282 (32.2%)	2343 (35.6%)	2903 (46.4%)	1283 (40.1%)	647 (40.8%)	650 (40.2%)	5483 (43.3%)
Black	244 (7.7%)	98 (6.9%)	53 (7.3%)	60 (8.1%)	455 (7.5%)	227 (7.4%)	102 (5.7%)	60 (7.0%)	43 (4.9%)	432 (6.6%)	471 (7.5%)	200 (6.3%)	113 (7.1%)	103 (6.4%)	887 (7.0%)
Asian	720 (22.6%)	325 (22.8%)	143 (19.7%)	149 (20.1%)	1337 (22.0%)	1342 (43.7%)	845 (47.6%)	378 (44.0%)	422 (48.2%)	2987 (45.4%)	2062 (33.0%)	1170 (36.6%)	521 (32.8%)	571 (35.3%)	4324 (34.2%)
Mixed	65 (2.0%)	25 (1.8%)	22 (3.0%)	18 (2.4%)	130 (2.1%)	64 (2.1%)	22 (1.2%)	14 (1.6%)	18 (2.1%)	118 (1.8%)	129 (2.1%)	47 (1.5%)	36 (2.3%)	36 (2.2%)	248 (2.0%)
Other	170 (5.3%)	79 (5.6%)	48 (6.6%)	41 (5.5%)	338 (5.6%)	136 (4.4%)	86 (4.8%)	40 (4.7%)	36 (4.1%)	298 (4.5%)	306 (4.9%)	165 (5.2%)	88 (5.5%)	77 (4.8%)	636 (5.0%)
Unknown	284 (8.9%)	194 (13.6%)	97 (13.3%)	105 (14.2%)	680 (11.2%)	102 (3.3%)	138 (7.8%)	85 (9.9%)	74 (8.5%)	399 (6.1%)	386 (6.2%)	332 (10.4%)	182 (11.5%)	179 (11.1%)	1079 (8.7%)
**Time since last screening test was due**															
6–24 months (Late)	1076 (33.7%)	448 (31.5%)	243 (33.4%)	220 (29.7%)	1987 (32.7%)	1100 (35.9%)	492 (27.7%)	201 (23.4%)	213 (24.3%)	2006 (30.5%)	2176 (34.8%)	940 (29.4%)	444 (28.0%)	433 (26.8%)	3993 (31.5%)
>24 months (Very late)	1477 (46.3%)	165 (11.6%)	67 (9.2%)	86 (11.6%)	1795 (29.5%)	1225 (39.9%)	163 (9.2%)	90 (10.5%)	88 (10.1%)	1566 (23.8%)	2702 (43.2%)	328 (10.3%)	157 (9.9%)	174 (10.8%)	3361 (26.6%)
Never screened	632 (19.8%)	810 (56.9%)	416 (57.2%)	435 (58.7%)	2293 (37.7%)	742 (24.2%)	1119 (63.1%)	568 (66.0%)	570 (65.1%)	2999 (45.6%)	1374 (22.0%)	1929 (60.3%)	984 (62.0%)	1005 (62.2%)	5292 (41.8%)
Missing	4 (0.1%)	0 (0.0%)	1 (0.1%)	0 (0.0%)	5 (0.1%)	1 (0.0%)	0 (0.0%)	1 (0.1%)	4 (0.5%)	6 (0.1%)	5 (0.1%)	0 (0.0%)	2 (0.1%)	4 (0.2%)	11 (0.1%)

Note percentages are column percentages.

In total, 516 women returned a self-sample. Of these, 15 were ineligible and 3 did not return a completed laboratory consent form or identifiers on the sample, so we do not know who the sample belonged to. Of the remaining 498, 465 were returned within 6 months. Overall, 150/1587 (9.5%) women requested a self-sampling kit after receiving a letter; 74 (49.3%) via the web and 76 (50.7%) by post. Almost a third of women who requested a kit by post were aged ≥45 years (31.6%), compared to 13.5% among those who requested a kit online (Table S1). No adverse events were reported.

### Effect of cluster randomisation

In the primary analysis, 5.6% (N = 342/6080) of women at GP practices randomised to opportunistically offer self-sampling kits returned a self-sample within 6 months, compared with 1.9% (N = 123/1735) in the no opportunistic offer arm (Table 2). After adjusting for age, ethnic background and screening history in the mixed effects model (Table S2), an additional 3.72% (95% CI: 3.13–4.31%) of individuals in practices randomised to the opportunistic offer arm returned a self-sample kit. The relative effect of being in the opportunistic offer arm was statistically significantly larger for women who were ‘late’ (OR = 1.73, 95% CI: 1.04–2.89) or ‘very late’ (OR = 2.88, 95% CI: 1.55–5.38) compared with women who had never been screened (Table S3), but the additive effect of randomisation in this model was more homogeneous: 4.55% (3.01–6.08%), 4.89% (3.38–6.41%), 3.33% (1.33–5.33%) among late, very late, and never screened, respectively (Table 3).

**Table 2:** Self-sampling uptake by cluster and individual-level randomisation.

Cluster randomisation:	Opportunistic offer of self-sampling kit					No opportunistic offer of self-sampling kit					Total				
Individual-level randomisation	Not randomised	No systematic offer	Sent letter	Sent kit	Total	Not randomised	No systematic offer	Sent letter	Sent kit	Total	Not randomised	No systematic offer	Sent letter	Sent kit	Total
No. women total	3189	1423	727	741	6080	3068	1774	860	875	6577	6257	3197	1587	1616	12,657
No. women offered at least one self-sample^a^	246	104	727	741	1818	0	0	860	875	1735	246	104	1587	1616	3553
No. women who returned self-sample within 6 months	137	54	45	106	342	0	0	31	92	123	137	54	76	198	465
% returned self-sample kit^b^	4.3%	3.8%	6.2%	14.3%	5.6%	0.0%	0.0%	3.6%	10.5%	1.9%	2.2%	1.7%	4.8%	12.3%	3.7%
% returned self-sample kit as a proportion of those offered a self-sample^c^	55.7%	51.9%	6.2%	14.3%	18.8%			3.6%	10.5%	7.1%	55.7%	51.9%	4.8%	12.3%	13.1%
No. women screened during study	856	286	173	231	1546	629	295	168	226	1319	1485	581	341	457	2865
% screened	26.8%	20.1%	23.8%	31.2%	25.4%	20.5%	16.6%	19.5%	25.8%	20.1%	23.7%	18.2%	21.5%	28.3%	22.6%

a Women either offered a kit opportunistically (i.e., by personal offer during a consultation) or systematically (by receiving letter inviting them to order a kit or receiving a kit in the post).

b The number (No.) of women who returned a self-sample kit within 6 months divided by the total number of women.

c The number of women who returned a self-sample kit within 6 months divided by the number of women either offered a kit opportunistically, sent a kit, or sent a letter inviting them to order a kit.

**Table 3:** Modelled absolute probability of returning a self-sample kit within 6 month, by screening history^a^ and cluster randomisation (GP^b^ practices offering opportunistic offer vs no opportunistic offer).

	No opportunistic offer	Opportunistic offer	Total	Absolute difference in uptake, opportunistic offer: no opportunistic offer
Late	1.52% (0.90%–2.13%)	5.42% (4.05%–6.78%)	3.45% (2.71%–4.20%)	4.55% (3.01%–6.08%)
Very late	0.85% (0.40%–1.31%)	5.05% (3.74%–6.35%)	2.94% (2.25%–3.62%)	4.89% (3.38%–6.41%)
Never	2.90% (2.08%–3.72%)	6.03% (4.61%–7.45%)	4.45% (3.63%–5.27%)	3.33% (1.33%–5.33%)
Total	1.87% (1.41%–2.34%)	5.56% (4.46%–6.65%)	3.72% (3.13%–4.31%)	4.40% (2.84%–5.95%)

a Screening history was defined as late (6–24 months overdue), very late (>24 months overdue, but screened previously) or never screened. Women aged <28 years who had never been screened were classified as ‘late’.

b General Practitioner.

### Uptake of opportunistic offers

Of 449 women known to have been opportunistically offered a self-sample kit, 333 (74.2%) accepted the offer, and 234 (52.1%, 95% CI: 47.5–56.7%) returned a sample within 6 months. Some of those who returned an opportunistic sample were also randomised to receive a kit (N = 49) or a letter (N = 48). Of the women in the opportunistic offer arm who were not individually randomised to be sent a letter or a kit, 4.1% (95% CI: 3.6–4.8%) returned a self-sample.

### Who was opportunistically offered self-sampling?

Over a mean of 11.1 months, 449 women cluster-randomised to ‘opportunistic offer’ were offered a self-sampling kit opportunistically, corresponding to 7.3% of overdue women in practices randomised to this option. There were significant differences in this proportion between GP practices (Χ^2^_5_ = 66.19, p < 0.001, range 4.0%–10.7%, Table S4). In a fully adjusted logistic regression model (Table S5), age (p < 0.001) and ethnicity (p = 0.001) were both significant predictors of being opportunistically offered self-sampling, but screening history was not (p = 0.122). Older women were more likely to be opportunistically offered a kit (OR = 2.64 (95% CI: 1.95–3.57 for aged 55–64 vs 25–34)), as were Asian (OR = 1.68, 95% CI: 1.31–2.16) and Black (OR = 1.50, 95% CI: 1.05–2.15) women compared with White women.

### Uptake by individual randomisation

Across all 13 GP practices, 198 of the 1616 women sent a kit (12.3%, 95% CI: 10.7–13.9%) and 76 of the 1587 women sent a letter inviting them to order a kit (4.8%, 95% CI: 3.8–6.0%) returned a self-sample within 6 months (Table 2). Of those individually randomised to no systematic offer, 1.7% (54/3197, 95% CI: 1.3–2.2%) returned a self-sampling within 6 months (They would have been returning a self-sample following an opportunistic offer). Both sending a letter and sending a kit resulted in higher uptake compared with no systematic offer: risk difference (RD using marginal estimates) = 10.5% (95% CI: 8.9–12.2%, p < 0.001) for kit and RD = 3.1% (95% CI: 2.0–4.2%, p < 0.001) for letter. The kit was significantly better than the letter (RD using marginal estimates = 7.5%, 95% CI: 5.6–9.4%). We note that the risk differences compared with randomised to no systematic offer were similar in opportunistic offer GP practices and no opportunistic offer GP practices (Table 2).

In comparison with the return percentage of 10.5% (92/875, 95% CI: 8.7–12.7%) among those sent a kit in the no opportunistic offer practices, uptake was 54.6% (191/350, 95% CI: 49.3–59.7%) among women known to have received an opportunistic offer (and not randomised to be mailed a self-sample kit or a letter).

In practices randomised to no opportunistic offers, White and Black women were most likely to return a sent kit (16.0% and 16.3%, respectively, Table S6), and uptake increased with increasing age (from 9.4% among women aged 25–34 to 15.2% aged 55–64 years). Uptake among women sent a letter offering the opportunity to order a kit was uniformly low across all ages, ethnic backgrounds and screening histories (0–5.3%). The proportion of women from practices randomised to opportunistic offering who returned a self-sampling kit increased with age, from 13.3% (95% CI: 11.1–15.8%) among women aged 25–34 years to 37.7% (30.9–44.8%) among women aged 55–64 years (Table S6).

### Sensitivity analyses

When looking at the impact of both the cluster randomisation and individual level randomisation, results were very similar in all the sensitivity analyses (Tables S7–S9).

### Follow-up of HPV-positive women

Of the 71 women who tested HPV positive on their self-sample, 51 (71.8%, 95% CI: 60.5–81.0%) either had a clinician-collected sample or attended colposcopy within 6 months (Table S10). None (0%, 95% CI: 0–5.2%) had CIN2+ recorded in their GP records. Although only 44.4% (N = 4/9) of those with a positive test who were sent a letter attended follow-up within 6 months (compared with 71.4% (N = 20/28) for those sent a kit and 79.4% (N = 27/34) for those with an opportunistic offer), the numbers were small and the difference is not statistically significant (p = 0.077).

### Women completing any cervical screening

The overall proportion of non-attenders screened during the study (either through routine screening or self-sampling) was higher in the opportunistic offer-arm GPs compared with the no opportunistic offer-arm GPs (25.4% vs 20.1%, p < 0.001, Table 2). Results from an adjusted mixed effects model (Table S11) showed that being in the opportunistic offer arm was associated with a 39% (95% CI: 13–70%) increase in odds of having any cervical screening during the study corresponding to a risk difference of 8.1% (95% CI: 3.8–12.5%). The additional percentage screened is similar to (slightly greater than) the additional percentage returning a self-sample implying that those who returned a self-sample would not have been screened otherwise. In the logistic model, there were statistically significant interactions between screening history and opportunistic offer, as well as screening history and individual-level randomisation (Table S12). The predicted percentage of women in each combination of screening history and opportunistic offer who had any cervical screening is shown in Table S13, using predictions from the model including interactions. However, the risk differences associated with the opportunistic offer were similar across screening history and individual randomisation (Table S13). Women who were sent a kit and in the opportunistic offer arm had an additional 16.2% (95% CI: 11.5–20.9%) chance of being screened compared with women individually randomised to no systematic offer and not in the opportunistic offer arm (32.7%–16.5%).

## Discussion

Our pragmatic randomised controlled trial compared different approaches for offering self-sampling kits to non-attenders, in a bid to assess the impact of each approach for increasing screening coverage. We showed that the uptake of self-sampling was by far the highest among women who were opportunistically offered kits in primary care. Although only women who had a consultation at their GP practice were able to benefit from this, previous work has shown that a median of 60% of non-attenders attend their GP practice over a 1-year period.^15^ Among women randomised to be mailed either a self-sampling kit or a letter inviting them to order a kit, those sent a kit had an additional 7.5% (=12.3%–4.8%) chance of returning a sample albeit with high levels of wastage. Compliance to follow-up amongst self-sample HPV positive was acceptable but noticeably lower than that observed in a recent implementation feasibility trial of self-sampling in London.^8^ Furthermore, compliance to follow-up was substantially lower in women who ordered a kit, although numbers were small. Overall, uptake was similar to that observed in our other two studies,^8^^,^^16^ indicating good external validity.

Self-sampling is highly acceptable to women.^16^^,^^20^^,^^21^ Previous work showed substantial variation in the uptake of an opportunistic offer, but results from this study are broadly in line with previous findings in three key areas. First, opportunistic uptake is substantially higher than other invitation modalities.^7^ Second, self-sampling uptake when directly sent a kit was higher than when sent an invitation to order a kit.^7^^,^^22^^,^^23^ Finally, uptake of direct mailout amongst non-attenders in the UK (8–12%) is modest in comparison to other European countries with similar healthcare systems.^9^^,^^10^^,^^14^^,^^22^, ^23^, ^24^

In our pilot study run over a similar number of months, 9% of women eligible for an opportunistic offer returned a self-sample kit,^16^ double the proportion in this study (4.1%). It was observed in both studies that the proportion of eligible patients offered self-sampling varied considerably between practices. Thus, the small number of practices offering self-sampling in each study may explain the differences. This study reports similar acceptance of an opportunistic offer to previous studies in England (74% of those opportunistically offered in this study, vs 68%^16^ and 85%^8^), and return of a self-sample among individuals who accepted an opportunistic offer (54% in this study vs 45%^16^ and 56%^8^). In YouScreen, a large study evaluating opportunistic offer of self-sampling in England,^8^ results from self-samples were included in the National Screening Programme call-recall database, and therefore contributed to the annual reward and incentive programme (Quality of Framework, QoF); this likely increased the GPs motivation to offer opportunistic screening to eligible women who attended for any reason.

Inviting women to order a self-sampling kit has been successful in the Capital Region of Denmark,^25^ but had limited success elsewhere.^26^^,^^27^ Direct kit mailout has been adopted in countries such as The Netherlands and Sweden. Lam et al. also found that mode of kit order for women invited to order kits was age dependent, with younger women tending to use online ordering compared to postal orders, suggesting both options for ordering kits should be offered.^28^

Among women who tested HPV-positive on a self-sample, compliance to follow up in this study was 72%, within the range observed in previous UK studies (59%–89%^8^^,^^9^^,^^16^), though lower than has been reported in studies in other countries (92% in a study in Denmark,^25^ 87% among overdue women mailed a kit in the US^29^^,^^30^).

A major strength of this trial is the design which enabled estimation of the impact of the opportunistic in-person offers (cluster randomised), over and above systematic postal modes of kit offer (individually randomised). It also allowed estimation of the proportion of the non-attenders who were offered a self-sampling kit opportunistically. Another strength relates to the pragmatic design and real-world setting of GP primary care, which promotes external validity and realistic uptake estimates.

A study limitation is the reliance on clinical codes in primary care records to determine eligibility and outcomes and to record randomisation. Cervical screening tests and colposcopy visits may be under-recorded in GP records leading to underestimates of attendance to follow-up. The COVID pandemic may have reduced attendance to follow up as access to GP appointments was limited. Furthermore, histology is poorly recorded in GP records meaning that some women diagnosed with CIN2+ may not have been reported in our study; assuming the same underlying CIN2+ rate as in YouScreen we would have expected 1.0% of screened women to have CIN2+.^8^ We also found a small number of randomised women were not coded as such in the records. We also note that the primary endpoint (returning a self-sample kit) was not defined for women who were cluster randomised to no opportunistic offer and were not individually randomised to be sent a letter or a kit, and was therefore not the ideal endpoint. However, since this was defined in the trial protocol and statistical analysis plan, this was the primary endpoint used in the study. To address this, we included a secondary endpoint of attending any cervical screening, which was appropriately defined for all individuals in the study.

Another limitation is that self-samples were not recorded as being part of the national screening programme, which we know from anecdotal GP feedback can disincentivize GPs from offering self-sampling due to perceived experimental nature and exclusion from financial reimbursement schemes. Indeed, the proportion of women opportunistically offered a self-sample was lower than the levels observed in our previous studies, and it proved challenging to extract primary care data for analysis. Additionally, translated invitational materials were not used in this study.

A key benefit of direct mail approaches is that kits are systematically distributed unbiasedly to all non-attenders meeting pre-determined criteria. By contrast, opportunistic offering relies both on non-attenders presenting to primary care and on healthcare professionals offering a kit. Nevertheless, in this study, opportunistic offers were more likely to be made to Black and Asian than to White women, and uptake of the offer was similar in all three ethnic groups. Although the opportunistic offer only reaches a subgroup of under-screened women, it provides an opportunity to ask questions, and could be supplemented with a systematic offer. Only 53% of women who ordered a self-sample kit and 54% of those who accepted an opportunistic offer returned a self-sample; this could be improved with reminder texts or postcards. Encouraging women who accept an opportunistic offer to collect the sample on-site may also increase the proportion of returned kits.

We have demonstrated that opportunistically offering self-sampling to cervical screening non-attenders when consulting in general practice (as prompted by an electronic popup) is an efficient and effective approach to increasing cervical screening participation. It also defied the ‘inverse care law’ whereby those who most need health care are least likely to receive it, in that it was successful across ethnic groups and even in women who had never been screened before. Notably, whereas few Black and Asian women returned a kit in response to receiving a letter, over half returned a sample following an opportunistic offer. Further almost half of those who returned a sample had never been screened before.

We recommend implementing opportunistic personal offers of self-sampling in the UK and in other countries that use primary care as the front door to health care, as is already done in Australia.^31^ In the UK, only a very small subgroup of adults never engage with GP primary care. Nevertheless, because GP offerings were heterogenous, systematic mail-out of kits should be considered in practices in which the opportunistic approach is not successful.

Further work is needed into how to reduce the attrition rates. Substantial numbers accepted (opportunistic offer) or ordered (sent letter) a kit but did not return a sample and over a quarter of those whose self-sample was HPV positive did not follow through with a clinician sample. Use of text reminders^32^ or even phone calls could be valuable. For instance, in the Capital Region of Denmark which offered self-sampling through letters in 2017–18, return-of-kit reminders generated 6.1% additional participation.^25^ In YouScreen,^8^ GP practices were asked to help women who tested HPV positive on a self-sample book their follow up test and compliance by the end of the trial was 89.1%.

Self-sampling implementation as an approach to increase coverage has provided cautionary tales in the transition from research to real-world settings.^33^ Uptake of self-sampling amongst the non-attendee population has been lower than hoped in The Netherlands.^34^ Further, results from the Netherlands suggest that in routine practice primary self-sampling may detect less high-grade CIN that clinician sampling.^35^

Offering self-sampling to those overdue cervical screening is an effective approach to enabling those individuals to participate in screening. Personal offers in GP primary care to patients consulting for a variety of reasons were more effective than the more usual approach of systematically offering self-sampling by post. However, only a small proportion of women overdue screening received such an offer over the course of a year. The secondary outcome suggests those screened by self-sampling would not have been screened otherwise. Offering self-sampling to those overdue cervical screening increases screening coverage and should result in lower rates of cervical cancer.

## Contributors

AL and PS designed the study concept and wrote the study protocol. RL, AL, and PS wrote the manuscript. BN wrote the statistical analysis plan. RL conducted the statistical analysis. PS oversaw the statistical analysis. RL, AL, and PS interpreted the data. JR acquired the data. All authors revised and approved the manuscript before submission. All authors read and approved the final version of the manuscript. AL and RL directly accessed and verified the underlying data used in this manuscript.

## Data sharing statement

Anonymous data generated by our research including the data used in this article will be made available, wherever legally and ethically required. Deidentified patient level data and the full dataset with low risk of identification and the data dictionary are available on reasonable request from the corresponding author after approval by the trial independent oversight committee and the Chief Investigator. The protocol is available at https://doi.org/10.1186/ISRCTN23940319.

## Declaration of interests

AL declares in-kind support from Copan Italia S.p.A in the form of provision of the 552C.80 FLOQSwab for the YouScreen study, received travel and accommodation to attend an expert meeting from Copan (December 2022), travel and accommodation provided by the meeting as a speaker at European Federation For Colposcopy meeting (October 2023), and travel and accommodation provided by the conference for the 2023 British Society for Colposcopy and Cervical Pathology meeting (May 2023). PS declares grants from North Central London Cancer Alliance and North East London Cancer Alliance, consulting fees from Roche for advisory board, participates on NIHR and IARC DMC/Advisory board; has received self-sampling kits from PreventX Limited for a previous trial, and has patents planned with Queen Mary Innovation. BN is the independent statistician on the data monitoring committees of several trials relating to cancer prevention. JR received travel reimbursement from Hologic Inc to attend a Patient Advocacy Group meeting. RL declares no conflicts of interest.

## Appendix A Supplementary data

Supplementary Results, Tables and Figure
