# Supplementary material for: Impact of mode of offer of self-sampling to people overdue cervical screening on screening participation: a randomised controlled trial
Source: eClinicalMedicine. 2025 Jul 28;86:103357. doi: 10.1016/j.eclinm.2025.103357 (PMC12399199; doi:10.1016/j.eclinm.2025.103357)

**Supplement to ‘****Impact of mode of offer of self-sampling to people overdue cervical screening on screening participation: a randomised controlled trial’**

Anita WW Lim* Rebecca Landy*, Jane Rigney, Bernard North, Peter D Sasieni

**Supplementary Results**

Table S1: Age distribution of individuals who requested a kit after being sent a letter, by mode of order

|  | Web | | Post | |
| --- | --- | --- | --- | --- |
| Age (years) | N | % | N | % |
| 25-34 | 38 | 51.4% | 32 | 42.1% |
| 35-44 | 26 | 35.1% | 20 | 26.3% |
| 45-54 | 4 | 5.4% | 11 | 14.5% |
| 55-64 | 6 | 8.1% | 13 | 17.1% |

Table S2: Results from a mixed effects regression model with the outcome of returning a self-sample within 6 months

|  | **OR (95% CI)** | **p-value** |
| --- | --- | --- |
| **Ethnicity** |  |  |
| White | Ref | 0.065 |
| Asian | 0.74 (0.58, 0.94) |  |
| Black | 0.88 (0.60, 1.30) |  |
| Mixed | 0.36 (0.13, 0.99) |  |
| Other | 0.89 (0.58, 1.38) |  |
| Unknown | 0.90 (0.65, 1.25) |  |
|  |  |  |
| **Age** |  |  |
| 25-34 | Ref | <0.001 |
| 35-44 | 1.07 (0.84, 1.36) |  |
| 45-54 | 1.23 (0.92, 1.63) |  |
| 55-64 | 2.32 (1.75, 3.07) |  |
|  |  |  |
| **Screening history** |  |  |
| Late | Ref | 0.001 |
| very late | 0.86 (0.66, 1.12) |  |
| Never | 1.33 (1.06, 1.66) |  |
|  |  |  |
| **Opportunistic offer** |  |  |
| No | Ref | <0.001 |
| Yes | 3.11 (2.24, 4.32) |  |
|  |  |  |
| Constant | 0.017 (0.012, 0.023) | <0.001 |

Table S3: Results from a mixed effects regression model with the outcome of returning a self-sample within 6 months, allowing for an interaction between screening history and GP-level randomisation

|  | **OR (95% CI)** |
| --- | --- |
| **Ethnicity** |  |
| White | Ref |
| Asian | 0.74 (0.58, 0.95) |
| Black | 0.90 (0.61, 1.33) |
| Mixed | 0.37 (0.14, 1.01) |
| Other | 0.89 (0.58, 1.37) |
| Unknown | 0.90 (0.65, 1.25) |
|  |  |
| **Age** |  |
| 25-34 | Ref |
| 35-44 | 1.07 (0.84, 1.35) |
| 45-54 | 1.23 (0.92, 1.64) |
| 55-64 | 2.34 (1.77, 3.10) |
|  |  |
| **Time since last screening test was due*** |  |
| 6-24 months | 0.51 (0.33, 0.80) |
| >24 months | 0.30 (0.17, 0.53) |
| Never screened | Ref |
|  |  |
| **GP-level randomisation** |  |
| Usual care | Ref |
| Opportunistic offer | 2.16 (1.47, 3.18) |
|  |  |
| Late*Opportunistic offer | 1.73 (1.04, 2.89) |
| Very late*Opportunistic offer | 2.88 (1.55, 5.38) |
|  |  |
| Constant | 0.028 (0.020, 0.039) |

*Screening history was defined as late (6-24 months overdue), very late (>24 months overdue, but screened previously) or never screened. Women aged <28 years who had never been screened were classified as ‘late’.

Table S4: Characteristics of women in the GP-level randomisation group of opportunistic offer who received an opportunistic offer, regardless of their individual-level randomisation.

|  | Opportunistically offered | | | |
| --- | --- | --- | --- | --- |
|  | Yes | | No | |
|  | N | % | No | % |
| **Total** | 449 | 7.3% | 5669 | 92.7% |
| **Age (years)** |  |  |  |  |
| 25-34 | 133 | 5.4% | 2343 | 94.6% |
| 35-44 | 126 | 6.4% | 1853 | 93.6% |
| 45-54 | 99 | 9.7% | 922 | 90.3% |
| 55-64 | 91 | 14.2% | 551 | 85.8% |
| Missing |  |  |  |  |
|  |  |  |  |  |
| **Ethnic background** |  |  |  |  |
| White | 225 | 7.1% | 2935 | 92.9% |
| Black | 40 | 8.8% | 415 | 91.2% |
| Asian | 116 | 8.6% | 1229 | 91.4% |
| Mixed | 9 | 6.9% | 122 | 93.1% |
| Other | 19 | 5.6% | 320 | 94.4% |
| Unknown | 40 | 5.8% | 645 | 94.2% |
|  |  |  |  |  |
| **Time since last screening test was due*** |  |  |  |  |
| 6-24 months | 132 | 6.6% | 1868 | 93.4% |
| >24 months | 172 | 9.5% | 1635 | 90.5% |
| Never screened | 144 | 6.2% | 2165 | 93.8% |
| Missing | 1 |  | 1 | 50.0% |
|  |  |  |  |  |
| **GP practice** |  |  |  |  |
| A | 80 | 9.5% | 764 | 90.5% |
| B | 68 | 4.0% | 1640 | 96.0% |
| C | 112 | 10.7% | 932 | 89.3% |
| D | 40 | 6.1% | 618 | 93.9% |
| E | 78 | 10.6% | 661 | 89.4% |
| F | 71 | 6.3% | 1054 | 93.7% |

Note percentages are row percentages. *Screening history was defined as late (6-24 months overdue), very late (>24 months overdue, but screened previously) or never screened. Women aged <28 years who had never been screened were classified as ‘late’.

Table S5: Results from a logistic regression model with the outcome of being opportunistically offered a self-sample kit when attending the GP for any reason

|  | **OR (95% CI)** | **p-value** |
| --- | --- | --- |
| **Age (years)** |  |  |
| 25-34 | 1 (Ref) | <0.001 |
| 35-44 | 1.12 (0.87, 1.45) |  |
| 45-54 | 1.72 (1.29, 2.29) |  |
| 55-64 | 2.64 (1.95, 3.57) |  |
|  |  |  |
| **Ethnic background** |  |  |
| White | 1 (Ref) | 0.001 |
| Black | 1.50 (1.05, 2.15) |  |
| Asian | 1.68 (1.31, 2.16) |  |
| Mixed | 1.14 (0.57, 2.30) |  |
| Other | 0.90 (0.55, 1.47) |  |
| Unknown | 0.90 (0.62, 1.29) |  |
|  |  |  |
| **Time since last screening test was due*** | | |
| 6-24 months | 1 (Ref) | 0.122 |
| >24 months | 1.20 (0.93, 1.55) |  |
| Never screened | 0.94 (0.73, 1.20) |  |
|  |  |  |
| **GP practice** | 1 (Ref) |  |
| A | 0.42 (0.30, 0.59) | <0.001 |
| B | 1.29 (0.95, 1.76) |  |
| C | 0.62 (0.41, 0.93) |  |
| D | 1.23 (0.88, 1.72) |  |
| E | 0.63 (0.45, 0.88) |  |
| F | 1 (Ref) |  |

*Screening history was defined as late (6-24 months overdue), very late (>24 months overdue, but screened previously) or never screened. Women aged <28 years who had never been screened were classified as ‘late’.

Table S6: Proportion of women who were offered at least one self-sample kit* who returned one within 6 months, by age, ethnic background and screening history

| Cluster randomisation: | Opportunistic offer of self-sampling kit | | | | | No opportunistic offer of self-sampling kit | | | | | Total | | | | |
| --- | --- | --- | --- | --- | --- | --- | --- | --- | --- | --- | --- | --- | --- | --- | --- |
| Individual-level randomization: | Not randomised | No systematic offer | Sent letter | Sent kit | Total | Not randomised | No systematic offer | Sent letter | Sent kit | Total | Not randomised | No systematic offer | Sent letter | Sent kit | Total |
| **Age (years)** |  |  |  |  |  |  |  |  |  |  |  |  |  |  |  |
| 25-34 | 56.0% (26/50) | 53.5% (23/43) | 4.6% (17/369) | 11.7% (45/385) | 13.3% (113/847) | 0 | 0 | 3.1% (14/458) | 9.4% (44/466) | 6.3% (58/924) | 56.0% (26/50) | 53.5% (23/43) | 3.7% (31/827) | 10.5% (89/851) | 9.7% (171/1771) |
| 35-44 | 57.5% (42/73) | 61.9% (13/21) | 7.9% (16/203) | 12.9% (26/202) | 19.4% (97/202) | 0 | 0 | 4.3% (10/232) | 10.2% (24/235) | 7.3% (34/467) | 57.5% (42/73) | 61.9% (13/21) | 6.0% (26/435) | 11.4% (50/437) | 13.6% (131/966) |
| 45-54 | 44.8% (26/58) | 44.0% (11/25) | 3.3% (3/92) | 17.3% (17/98) | 20.9% (57/273) | 0 | 0 | 3.8% (4/106) | 13.0% (14/108) | 8.4% (18/214) | 44.8% (26/58) | 44.0% (11/25) | 3.5% (7/198) | 15.0% (31/206) | 15.4% (75/487) |
| 55-64 | 63.1% (41/65) | 46.7% (7/15) | 14.3% (9/63) | 32.1% (18/56) | 37.7% (75/199) | 0 | 0 | 4.7% (3/64) | 15.2% (10/66) | 10.0% (13/130) | 63.1% (41/65) | 46.7% (7/15) | 9.4% (12/127) | 23.0% (28/122) | 26.7% (88/329) |
| Missing |  |  |  |  |  |  |  |  |  |  |  |  |  |  |  |
| **Ethnic background** |  |  |  |  |  |  |  |  |  |  |  |  |  |  |  |
| White | 56.5% (74/131) | 55.1% (27/49) | 8.0% (29/49) | 15.8% (58/368) | 20.6% (188/912) | 0 | 0 | 5.3% (15/283) | 16.0% (45/282) | 10.6% (60/565) | 56.5% (74/131) | 55.1% (27/49) | 6.8% (44/647) | 15.8% (103/650) | 16.8% (248/1473) |
| Black | 55.0% (11/20) | 50.0% (4/8) | 7.5% (4/53) | 8.3% (5/60) | 17.0% (24/141) | 0 | 0 | 0.0% (0/60) | 16.3% (7/60) | 6.8% (7/103) | 55.0% (11/20) | 50.0% (4/8) | 3.5% (4/113) | 11.7% (12/103) | 12.7% (31/244) |
| Asian | 50.8% (32/63) | 45.2% (14/31) | 2.8% (4/143) | 12.1% (18/149) | 17.6% (68/386) | 0 | 0 | 2.6% (10/378) | 6.4% (27/378) | 4.6% (37/800) | 50.8% (32/63) | 45.2% (14/31) | 2.7% (14/521) | 7.9% (45/571) | 8.9% (105/1186) |
| Mixed | 0.0% (0/3) | 0.0% (0/1) | 9.1% (2/22) | 5.6% (1/18) | 6.8% (3/44) | 0 | 0 | 0.0% (0/14) | 5.6% (1/14) | 3.1% (1/32) | 0.0% (0/3) | 0.0% (0/1) | 5.6% (2/36) | 5.6% (2/36) | 5.3% (4/76) |
| Other | 44.4% (4/9) | 80.0% (4/5) | 2.1% (1/48) | 24.4% (10/41) | 18.4% (19/103) | 0 | 0 | 5.0% (2/40) | 8.3% (3/40) | 6.6% (5/76) | 44.4% (4/9) | 80.0% (4/5) | 3.4% (3/88) | 16.9% (13/77) | 13.4% (24/179) |
| Unknown | 80.0% (16/20) | 50.0% (5/10) | 5.2% (5/97) | 13.3% (14/105) | 17.2% (40/232) | 0 | 0 | 4.7% (4/85) | 12.2% (9/85) | 8.2% (13/159) | 80.0% (16/20) | 50.0% (5/10) | 4.9% (9/182) | 12.8% (23/179) | 14.1% (53/391) |
| Mixed/Other/Unknown |  |  |  |  |  |  |  |  |  |  |  |  |  |  |  |
| **Time since last screening test was due**** | |  |  |  |  |  |  |  |  |  |  |  |  |  |  |
| 6-24 months | 67.7% (44/65) | 58.8% (20/34) | 6.6% (16/34) | 11.4% (25/220) | 18.7% (105/562) | 0 | 0 | 2.5% (5/201) | 10.8% (23/213) | 6.8% (28/414) | 67.7% (44/65) | 58.8% (20/34) | 4.7% (21/444) | 11.1% (48/433) | 13.6% (133/976) |
| >24 months | 52.7% (79/150) | 38.5% (5/13) | 11.9% (8/67) | 12.8% (11/86) | 32.6% (103/316) | 0 | 0 | 3.3% (3/90) | 13.6% (12/88) | 8.4% (15/178) | 52.7% (79/150) | 38.5% (5/13) | 7.0% (11/157) | 13.2% (23/174) | 23.9% (118/494) |
| Never screened | 43.3% (13/30) | 50.9% (29/57) | 5.0% (21/416) | 16.1% (70/435) | 14.2% (133/938) | 0 | 0 | 4.0% (23/568) | 9.5% (54/570) | 6.8% (77/1138) | 43.3% (13/30) | 50.9% (29/57) | 4.5% (44/984) | 12.3% (124/1005) | 10.1% (210/2076) |

* Women either offered a kit opportunistically (i.e. by personal offer during a consultation) or systematically (by receiving letter inviting them to order a kit or receiving a kit in the post. **Screening history was defined as late (6-24 months overdue), very late (>24 months overdue, but screened previously) or never screened. Women aged <28 years who had never been screened were classified as ‘late’.

Table S7: The number of women in each cluster-randomised group and individually-randomised group, the number of these women who were offered at least one self-sampling kit*, and the number of women who returned a self-sample kit within 6 months, using the most intensive randomisation** for the individual-level randomisation

| Cluster randomisation: | Opportunistic offer of self-sampling kit | | | | | No opportunistic offer of self-sampling kit | | | | | Total | | | | |
| --- | --- | --- | --- | --- | --- | --- | --- | --- | --- | --- | --- | --- | --- | --- | --- |
| Individual-level randomisation | Not randomised | No systematic offer | Sent letter | Sent kit | Total | Not randomised | No systematic offer | Sent letter | Sent kit | Total | Not randomised | No systematic offer | Sent letter | Sent kit | Total |
| No. women total | 3189 | 1423 | 696 | 772 | 6080 | 3068 | 1774 | 806 | 929 | 6577 | 6257 | 3197 | 1502 | 1701 | 12657 |
| No. women offered at least one self-sample* | 246 | 104 | 696 | 772 | 1818 | 0 | 0 | 806 | 929 | 1735 | 246 | 104 | 1502 | 1701 | 3553 |
| No. women who returned self-sample within 6 months | 137 | 54 | 43 | 108 | 342 | 0 | 0 | 27 | 96 | 123 | 137 | 54 | 70 | 204 | 465 |
| % of women who returned a self-sample within 6 months | 55.7% | 51.9% | 6.2% | 14.0% | 18.8% | - | - | 3.3% | 10.3% | 7.1% | 55.7% | 51.9% | 4.7% | 12.0% | 13.1% |

* Women either offered a kit opportunistically (i.e. by personal offer during a consultation) or systematically (by receiving letter inviting them to order a kit or receiving a kit in the post.** Being sent a self-sample kit is more intensive than being sent a letter to order a kit, which is more intensive than being in the no systematic offer arm

Table S8: The number of women in each cluster-randomised group and individually-randomised group, the number of these women who were offered at least one self-sampling kit*, and the number of women who returned a self-sample kit within 6 months, 8 months, and ever, using the first randomisation for the individual-level randomisation

| Cluster randomisation: | Opportunistic offer of self-sampling kit | | | | | Usual care | | | | | Total | | | | |
| --- | --- | --- | --- | --- | --- | --- | --- | --- | --- | --- | --- | --- | --- | --- | --- |
| Individual-level randomisation | Not randomised | No systematic offer | Sent letter | Sent kit | Total | Not randomised | No systematic offer | Sent letter | Sent kit | Total | Not randomised | No systematic offer | Sent letter | Sent kit | Total |
| N women total | 3189 | 1423 | 727 | 741 | 6080 | 3068 | 1774 | 860 | 875 | 6577 | 6257 | 3197 | 1587 | 1616 | 12657 |
| N women offered at least one self-sample* | 246 | 104 | 727 | 741 | 1818 | 0 | 0 | 860 | 875 | 1735 | 246 | 104 | 1587 | 1616 | 3553 |
| N women who returned self-sample within 4 months | 136 | 53 | 38 | 92 | 319 | 0 | 0 | 25 | 75 | 100 | 136 | 53 | 63 | 167 | 419 |
| % returned self-sample kit within 4 months | 4.3% | 3.7% | 5.2% | 12.4% | 5.2% | 0.0% | 0.0% | 2.9% | 8.6% | 1.5% | 2.2% | 1.7% | 4.0% | 10.3% | 3.3% |
| N women who returned self-sample within 8 months | 137 | 55 | 47 | 110 | 349 | 0 | 0 | 33 | 95 | 128 | 137 | 55 | 80 | 205 | 477 |
| % returned self-sample kit within 8 months | 4.3% | 3.9% | 6.5% | 14.8% | 5.7% | 0.0% | 0.0% | 3.8% | 10.9% | 1.9% | 2.2% | 1.7% | 5.0% | 12.7% | 3.8% |
| N women who returned self-sample | 137 | 55 | 54 | 118 | 364 | 0 | 0 | 35 | 99 | 134 | 137 | 55 | 89 | 217 | 498 |
| % returned self-sample kit | 4.3% | 3.9% | 7.4% | 15.9% | 6.0% | 0.0% | 0.0% | 4.1% | 11.3% | 2.0% | 2.2% | 1.7% | 5.6% | 13.4% | 3.9% |

* Women either offered a kit opportunistically (i.e. by personal offer during a consultation) or systematically (by receiving letter inviting them to order a kit or receiving a kit in the post.

Table S9: The number of women in each cluster-randomised group and individually-randomised group, the number of these women who were offered at least one self-sampling kit*, and the number of women who returned a self-sample kit within 6 months, using the first randomisation for the individual-level randomisation, excluding 41 individuals who were individually randomised more than once.

| Cluster randomisation: | Opportunistic offer of self-sampling kit | | | | | No opportunistic offer of self-sampling kit | | | | | | Total | | | | |
| --- | --- | --- | --- | --- | --- | --- | --- | --- | --- | --- | --- | --- | --- | --- | --- | --- |
| Individual-level randomisation | Not randomised | No systematic offer | Sent letter | Sent kit | Total | Not randomised | No systematic offer | Sent letter | Sent kit | Total | Not randomised | | No systematic offer | Sent letter | Sent kit | Total |
| N women total | 3189 | 1423 | 722 | 728 | 6062 | 3068 | 1774 | 853 | 859 | 6554 | 6257 | | 3197 | 1575 | 1587 | 12616 |
| N women offered at least one self-sample* | 246 | 104 | 722 | 728 | 1800 | 0 | 0 | 853 | 859 | 1712 | 246 | | 104 | 1575 | 1587 | 3512 |
| N women who returned self-sample within 6 months | 137 | 54 | 40 | 97 | 328 | 0 | 0 | 26 | 77 | 103 | 137 | | 54 | 66 | 174 | 431 |
| % returned self-sample kit | 4.3% | 3.8% | 5.5% | 13.3% | 5.4% | 0.0% | 0.0% | 3.0% | 9.0% | 1.6% | 2.2% | | 1.7% | 4.2% | 11.0% | 3.4% |
| N women screened within 6 months | 856 | 286 | 168 | 218 | 1546 | 629 | 295 | 161 | 210 | 1319 | 1485 | | 581 | 329 | 428 | 2865 |
| % screened | 26.8% | 20.1% | 23.3% | 29.9% | 25.5% | 20.5% | 16.6% | 18.9% | 24.4% | 20.1% | 23.7% | | 18.2% | 20.9% | 27.0% | 22.7% |

* Women either offered a kit opportunistically (i.e. by personal offer during a consultation) or systematically (by receiving letter inviting them to order a kit or receiving a kit in the post.

Table S10: Characteristics of the women who tested HPV positive on their self-sample, and the percentage who attended follow-up within 6 months

|  | Total number of self-sample HPV positive women | Percent who attend follow-up within 6 months (N) |  |
| --- | --- | --- | --- |
| Total | 71 | 51 | 71.8% |
| **Age (years)** |  |  |  |
| 25-34 | 34 | 26 | 76.5% |
| 35-44 | 20 | 12 | 60.0% |
| 45-54 | 8 | 6 | 75.0% |
| 55-64 | 9 | 7 | 77.8% |
| **Ethnic background** |  |  |  |
| White | 40 | 29 | 72.5% |
| Black | 7 | 5 | 71.4% |
| Asian | 10 | 8 | 80.0% |
| Mixed | 1 | 0 | 0.0% |
| Other | 4 | 4 | 100.0% |
| Unknown | 9 | 5 | 55.6% |
| **Time since last screening test was due*** |  |  |  |
| 6-24 months | 21 | 17 | 81.0% |
| >24 months | 16 | 13 | 81.3% |
| Never screened | 34 | 21 | 61.8% |
| **Mode of offer**** |  |  |  |
| Sent letter | 9 | 4 | 44.4% |
| Sent kit | 28 | 20 | 71.4% |
| Opportunistic offer | 34 | 27 | 79.4% |

*Screening history was defined as late (6-24 months overdue), very late (>24 months overdue, but screened previously) or never screened. Women aged <28 years who had never been screened were classified as ‘late’. **Sent letter: 4 women tested positive in the opportunistic offer arm, of whom 2 (50%) attended follow-up within 6-months; 5 tested positive of whom 2 (40%) attended follow-up within 6 months in the no opportunistic offer arm. Sent kit: 14 women tested positive in the opportunistic offer arm, of whom 10 (71%) attended follow-up within 6-months; 14 tested positive of whom 4 (29%) attended follow-up within 6 months in the no opportunistic offer arm.

Table S11: Results from a mixed effects regression model with the outcome of any screening during the study period

|  | OR (95% CI) | p-value |
| --- | --- | --- |
| **Ethnicity** |  |  |
| White | Ref | <0.001 |
| Asian | 1.14 (1.03, 1.27) |  |
| Black | 1.02 (0.86, 1.22) |  |
| Mixed | 0.87 (0.63, 1.20) |  |
| Other | 1.21 (1.00, 1.47) |  |
| Unknown | 0.74 (0.62, 0.89) |  |
|  |  |  |
| **Age (years)** |  |  |
| 25-34 | Ref | <0.001 |
| 35-44 | 1.18 (1.06, 1.30) |  |
| 45-54 | 1.00 (0.87, 1.14) |  |
| 55-64 | 0.84 (0.71, 0.99) |  |
|  |  |  |
| **Time since last screening test was due*** |  |  |
| 6-24 months | 1.57 (1.42, 1.74) | <0.001 |
| >24 months | 0.94 (0.83, 1.06) |  |
| Never screened | Ref |  |
|  |  |  |
| **Opportunistic offer** |  |  |
| No | Ref | 0.002 |
| Yes | 1.39 (1.13, 1.70) |  |
|  |  |  |
| **Individual randomisation** |  |  |
| No systematic offer | Ref | <0.001 |
| Sent letter | 1.24 (1.07, 1.44) |  |
| Sent kit | 1.79 (1.55, 2.07) |  |
| Not randomised | 1.34 (1.20, 1.51) |  |
|  |  |  |
| Constant | 0.16 (0.14, 0.20) | <0.001 |

*Screening history was defined as late (6-24 months overdue), very late (>24 months overdue, but screened previously) or never screened. Women aged <28 years who had never been screened were classified as ‘late’.

Table S12: Results from a mixed effects regression model with the outcome of having any cervical screening during the study

|  | **OR (95% CI)** |
| --- | --- |
| **Ethnicity** |  |
| White | Ref |
| Asian | 1.15 (1.04, 1.28) |
| Black | 1.03 (0.87, 1.23) |
| Mixed | 0.89 (0.64, 1.22) |
| Other | 1.20 (0.99, 1.46) |
| Unknown | 0.75 (0.62, 0.89) |
|  |  |
| **Age (years)** |  |
| 25-34 | Ref |
| 35-44 | 1.12 (1.01, 1.24) |
| 45-54 | 0.96 (0.84, 1.10) |
| 55-64 | 0.82 (0.70, 0.97) |
|  |  |
| **Time since last screening test was due*** |  |
| 6-24 months (late) | 0.91 (0.73, 1.14) |
| >24 months (very late) | 0.80 (0.57, 1.12) |
| Never screened | Ref |
|  |  |
| **Opportunistic offer** |  |
| No | Ref |
| Yes | 1.22 (0.97, 1.54) |
|  |  |
| **Individual randomisation** |  |
| No systematic offer |  |
| Sent letter | 1.26 (1.04, 1.52) |
| Sent kit | 1.75 (1.46, 2.09) |
| Not randomised | 0.74 (0.61, 0.90) |
|  |  |
| Late*Opportunistic offer | 1.26 (1.03, 1.54) |
| Very late*Opportunistic offer | 1.26 (1.01, 1.58) |
|  |  |
| Late*sent letter | 0.91 (0.65, 1.28) |
| Late*sent kit | 1.01 (0.73, 1.39) |
| Late*not randomised | 3.00 (2.29, 3.93) |
| Very late*sent letter | 1.05 (0.62, 1.76) |
| Very late*sent kit | 1.06 (0.66, 1.71) |
| Very late*not randomised | 1.62 (1.13, 2.33) |
|  |  |
| Constant | 0.20 (0.17, 0.25) |

*Screening history was defined as late (6-24 months overdue), very late (>24 months overdue, but screened previously) or never screened. Women aged <28 years who had never been screened were classified as ‘late’.

Table S13: Modelled absolute probability of any screening by the end of the study, by screening history and cluster randomisation (GP practices offering opportunistic offer vs no opportunistic offer)

|  | No opportunistic offer | Opportunistic offer | Total | Difference in screening between Opportunistic offer and No opportunistic offer |
| --- | --- | --- | --- | --- |
| Not randomised | 19.83% (17.52% - 22.13%) | 25.73% (22.97% - 28.49%) | 22.73% (20.84% - 24.62%) | 8.80% (4.43% - 13.16%) |
| No systematic offer | 16.49% (14.19% - 18.79%) | 21.51% (18.59% - 24.44%) | 18.94% (16.83% - 21.04%) | 6.70% (2.55% - 10.88%) |
| Sent letter | 19.56% (16.56% - 22.56%) | 25.22% (21.49% - 28.95%) | 22.32% (19.44% - 25.20%) | 7.05% (2.63% - 11.47%) |
| Sent kit | 25.89% (22.43% - 29.34%) | 32.66% (28.56% - 36.75%) | 29.20% (26.04% - 32.36%) | 7.93% (3.07% - 12.78%) |
| Total | 20.54% (18.31% - 22.78%) | 26.41% (23.68% - 29.13%) | 23.41% (21.65% - 25.16%) | 8.13% (3.76% - 12.49%) |

Figure S1: Timing of the intervention and the context of the English call/recall cervical screening programme


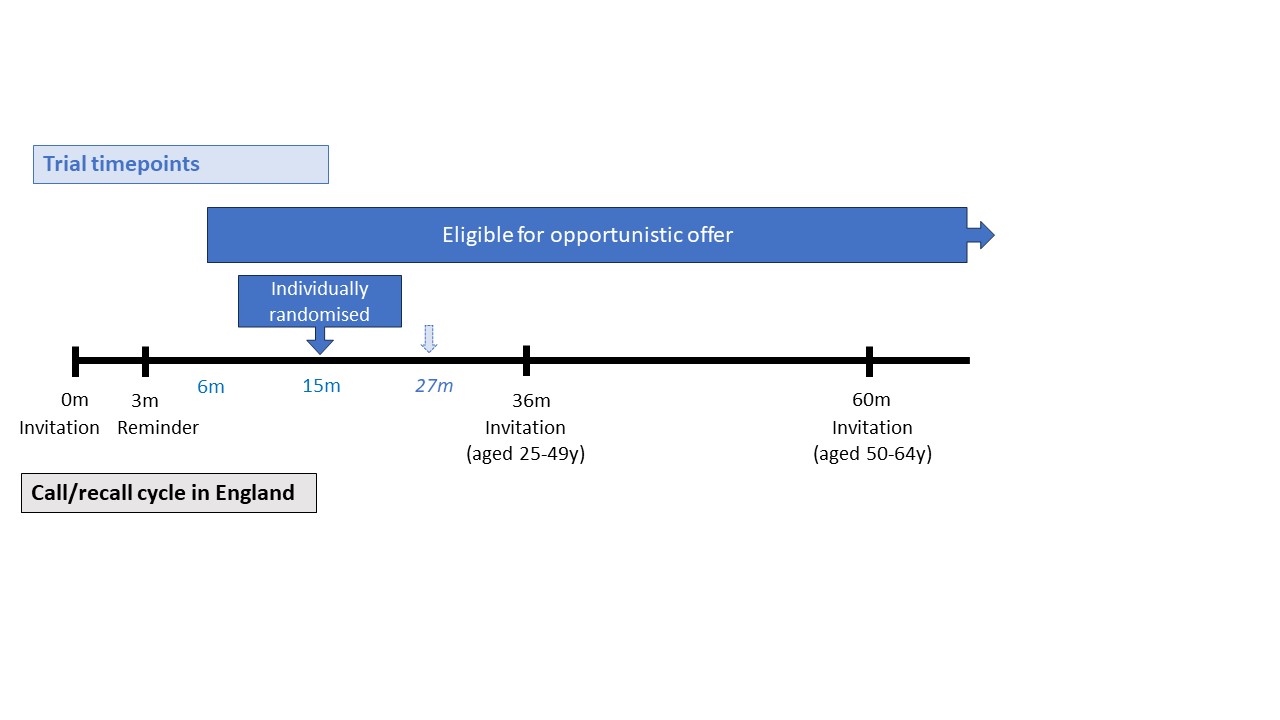

Supplement: Supplementary Results, Tables and Figure [file mmc1.docx]
